# Novel plasmid pCM3 harboring the *aph(3)* gene confers phosphorylation‐driven streptomycin resistance in *Clavibacter michiganensis*

**DOI:** 10.1002/mlf2.70091

**Published:** 2026-06-24

**Authors:** Xiaoli Xu, Cen Qian, Zhigang Hao, Meng Xie, Na Jiang, Jianqiang Li, Laixin Luo

**Affiliations:** ^1^ State Key Laboratory of Agricultural and Forestry Biosecurity, MARA Key Laboratory of Surveillance and Management for Plant Quarantine Pests, Beijing Key Laboratory of Seed Disease Testing and Control, College of Plant Protection China Agricultural University Beijing China; ^2^ Shanghai Key Laboratory of Agricultural Genetics and Breeding, Biotechnology Research Institute Shanghai Academy of Agricultural Sciences Shanghai China; ^3^ Xinjiang Key Laboratory of Agricultural Biosafety, Institute of Plant Protection Xinjiang Academy of Agricultural Sciences Urumqi China

## Abstract

Antibiotic resistance in plant pathogens threatens sustainable crop protection, and yet, its molecular basis remains incompletely understood. We report that streptomycin resistance in *Clavibacter michiganensis* strain TX‐0702 is mediated by a plasmid‐borne aminoglycoside phosphotransferase, APH(3), encoded on an uncharacterized plasmid, pCM3. Functional and biochemical analyses demonstrate that APH(3) inactivates streptomycin through phosphorylation, establishing a phosphorylation‐driven resistance mechanism in Gram‐positive plant pathogens. Sequence analyses reveal that pCM3 carries mobile genetic elements, suggesting environmental dissemination of resistance genes in plant‐associated microbes. These findings expand understanding of phytopathogen antibiotic resistance and plant microbiomes as potential resistance gene sources for agricultural, food, and human health safety.

Tomato bacterial canker, caused by *Clavibacter michiganensis*, is a destructive vascular disease that threatens global tomato production and seed trade.The pathogen is primarily transmitted through contaminated seeds, enabling long‐distance dissemination and persistent field outbreaks^1^. In the absence of commercially available resistant cultivars, disease management relies largely on quarantine measures, seed treatments, and chemical control strategies^2^. Among these, antibiotic‐based treatments remain effective once infection is established. However, the frequent application of antibiotics has raised concerns about the emergence and spread of resistant strains^3^.

Streptomycin, a prominent aminoglycoside antibiotic, has been widely used in the management of plant disease and the treatment of human diseases, including tuberculosis. It inhibits bacterial growth by impeding protein synthesis through its interaction with the ribosome^4^. Decades of research have elucidated that bacterial resistance to streptomycin is primarily achieved via two mechanisms. The first involves mutations in ribosomal components, particularly in the *rpsL* or 16S rRNA gene (*rrs*), which disrupt antibiotic binding to the ribosome and often confer stable, high‐level resistance in bacteria^5^. Additionally, mutations occurring in ribosomal RNA modification genes, such as *gidB*, have been reported to confer low‐level resistance in several bacterial species^6^. A second well‐established resistance strategy involves enzymatic modification of streptomycin, resulting in antibiotic inactivation^7^. Aminoglycoside‐modifying enzymes, including phosphotransferases and adenylyltransferases, function as self‐defense mechanisms in the producing organisms, and catalyze the transfer of a phosphate group to specific hydroxyl moieties on the streptomycin molecule, thereby inactivating it. Related resistance genes, such as *strAB* and *aadA*, are frequently found in mobile genetic elements and can spread across bacterial populations through horizontal gene transfer (HGT)^8^, ^9^.

Recent investigations have revealed that *C. michiganensis* shows significant streptomycin resistance. Field isolates carrying mutations at codon 43 of the *rpsL* gene display high levels of resistance^10^. A previous study of our group demonstrated that exposure of *C. michiganensis* strains to elevated streptomycin concentrations successfully induced high‐level resistance. This induced resistance was also attributed to mutations at codon 43 of the *rpsL* gene^11^. Overexpression of the mutated *rpsL* gene in streptomycin‐sensitive strains resulted in intermediate‐level resistance^11^. In contrast, wild‐type resistant strains, such as TX‐0702, which were isolated from diseased tomato fields, show resistance that is not mediated by mutations in the *rpsL*, *gidB*, *rrs*, or *strB* genes.

The present study aimed to determine the genetic basis of the unusually high‐level streptomycin resistance observed in the field‐derived *C. michiganensis* strain TX‐0702. Random transposon mutagenesis generated 30 streptomycin‐sensitive mutants, among which 15 showed markedly reduced resistance based on minimum inhibitory concentrations (MIC) assays (≤16 μg/ml) and were selected for the subsequent identification of transposon insertion sites. The remaining mutants retained wild‐type resistance levels (Table S1).

Inverse PCR, followed by sequencing, was performed on the 18 selected strains to determine the genetic lesion responsible for the loss of streptomycin resistance in these mutants. Thirteen distinct transposon insertion sites were identified. Several insertions mapped to annotated chromosomal loci in the TX‐0702 genome; however, subsequent functional analyses did not support a role for these genes in streptomycin resistance (Table S2). In contrast, seven insertion sequences could not be assigned to the reference genome or public databases. Assembly of overlapping fragments from these unmapped insertions yielded a contiguous 2739 bp sequence containing a 756 bp open reading frame encoding an aminoglycoside 3′ phosphotransferase, hereafter designated *aph(3)*. Third‐generation *de novo* genome sequencing was performed to localize the *aph(3)* in *C. michiganensis* TX‐0702. The assembly revealed a previously uncharacterized 51,235 bp plasmid, designated pCM3, in addition to the known plasmids pCM1 and pCM2. The Suk sequence, which was identified by inverse PCR and contains *aph(3)*, was localized to pCM3 (Figure 1A). A comparative analysis revealed that pCM3 shows 90.52% nucleotide identity (with 67% coverage) to plasmid D26b from *Curtobacterium* sp. MCBD17_026 (CP126283.1), while an ~6.6 kb region displayed near‐identical similarity to the *C. michiganensis* genome, suggesting a mosaic origin (Figure S1). Annotation of pCM3 identified multiple mobility‐associated genes, indicating that *aph(3)* is carried on a potentially mobilizable plasmid.

**Figure 1:**
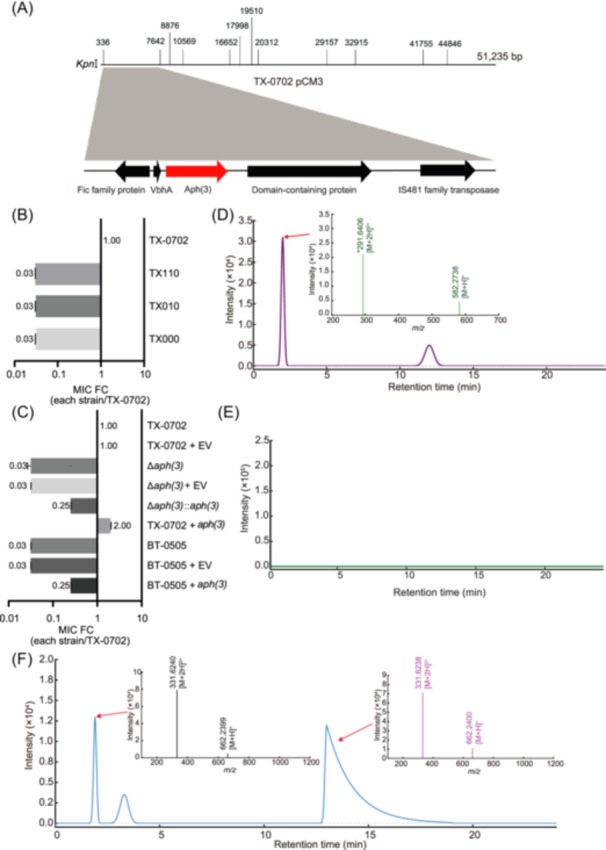
Identification of *aph(3)* and its role in streptomycin resistance in *Clavibacter michiganensis*. (A) Physical map of the *aph(3)* gene derived from *de novo* sequencing data, showcasing its genomic location, structural organization, and potential regulatory elements. (B) Minimum inhibitory concentrations (MICs) of streptomycin against plasmid‐cured strains of *C. michiganensis*, normalized to wild‐type TX‐0702. The TX110 lacks pCM3, TX010 lacks both pCM1 and pCM3, and TX000 lacks all three plasmids (pCM1, pCM2, and pCM3). Data are shown as mean ± SD (*n* = 3). (C) MICs of streptomycin against *C. michiganensis* strains with different levels of *aph(3)* expression, normalized to wild‐type TX‐0702. BT‐0505, streptomycin‐sensitive field strain. EV, empty vector control. Data are shown as mean ± SD (*n* = 3). (D) Liquid chromatography‐mass spectrometry (LC‐MS) analysis of streptomycin standard (20 μg/ml), showing a distinct retention time at ~2 min. The detected ions include the protonated molecular [M + H]^+^ at *m*/*z* 582.27 and the doubly charged ion [M + 2H]^2+^ at *m*/*z* 291.64. (E) Extracted ion chromatogram (EIC) of APH(3)‐treated samples scanned at *m*/*z* 582.27, showing complete loss of native streptomycin ions under this specific detection window. (F) LC‐MS detection of phosphorylated streptomycin in APH(3)‐treated samples. Peaks at *m*/*z* 662.24 ([M + H]^+^) and 331.62 ([M + H]^2+^) correspond to the monophosphorylated product. FC, fold change.

To validate the role of *aph(3)* and the plasmid pCM3 in streptomycin resistance, a series of plasmid‐cured *C. michiganensis* TX‐0702 derivatives were analyzed. The strain TX110 lacked pCM3, TX010 lacked both pCM1 and pCM3, and TX000 lacked of all three plasmids (pCM1, pCM2, and pCM3). MIC assays revealed that, while the MIC of the wild‐type TX‐0702 strain was 128 μg/ml, the MIC of all pCM3‐deficient strains was significantly reduced to 4 μg/ml (fold change [FC] = 0.03), indicating that pCM3 is essential for resistance (Figure 1B). This finding was corroborated by disc diffusion assays, in which the wild TX‐0702 strain was the only one to show no inhibition zone upon exposure to 1 mg/ml streptomycin, whereas the plasmid‐cured strains formed clear inhibition zones (~1.5 cm), indicating increased sensitivity (Figure S2A).

To further confirm the contribution of *aph(3)*, a gene knockout mutant Δ*aph(3)* was generated and its resistance phenotype was assessed alongside complemented Δ*aph(3)*::*aph(3)* and overexpression strains TX‐0702 + *aph(3)* and BT‐0505 + *aph(3)*. qPCR analysis confirmed differential *aph(3)* expression across these strains (Figure S2B). As shown in Figure 1C, Δ*aph(3)* showed a markedly reduced MIC of 4 μg/ml (FC = 0.03), similar to the sensitive field strain *C. michiganensis* BT‐0505. Complementation partially restored resistance, with Δ*aph(3)*::*aph(3)* reaching an MIC of 32 μg/ml (FC = 0.25), while overexpression of *aph(3)* in TX‐0702 further elevated the MIC to 256 μg/ml (FC = 2.00). The introduction of *aph(3)* into the sensitive strain BT‐0505 led to a significant increase in its streptomycin resistance level, confirming the functional contribution of *aph(3)*. Filter disc assays demonstrated that streptomycin pretreated with APH(3) lost its inhibitory activity against all *C. michiganensis* strains, indicating that APH(3) enzymatically inactivates the antibiotic. In contrast, the streptomycin‐sensitive strains Δ*aph(3)* and BT‐0505 showed clear inhibition zones (~2.0 ± 0.1 cm) when treated with unmodified streptomycin, while no inhibition was observed upon exposure to phosphorylated streptomycin (Figure S2C).

In order to characterize the enzymatic modification of streptomycin by APH(3) at the molecular level, liquid chromatography‐mass spectrometry (LC‐MS) analysis was performed. Purified recombinant APH(3) was incubated with streptomycin, followed by LC‐MS analysis. The standard streptomycin sample produced a signal, symmetrical LC‐MS peak at around 2 min. The [M + H]^+^ ion appeared at *m*/*z* of 582.27, consistent with its molecular weight (581.57), along with a doubly charged ion at *m*/*z* 291.64 (Figure 1D). However, this peak at *m*/*z* 582.27 was absent in the APH(3)‐treated samples (Figure 1E). In contrast, the result in Figure 1F revealed a new ion peak at *m*/*z* 662.24, which is consistent with the predicted mass of monophosphorylated streptomycin (C_21_H_40_N_7_O_15_P), accompanied by a doubly charged ion at *m*/*z* 331.62 (Figure 1F). In the distilled water‐treated control, only native streptomycin ions were observed at *m*/*z* 582.27 and 291.64, with a retention time of ~2 min (Figure S3A). No phosphorylated streptomycin peaks were detected in this negative control (Figure S3B).

In this study, we elucidated a novel mechanism of streptomycin resistance in *C. michiganensis* through transposon insertion mutagenesis and inverse PCR. Previous approaches, including resequencing, had failed to identify streptomycin resistance determinants in strain TX‐0702, highlighting the need for alternative strategies^11^, ^12^. By constructing a library of streptomycin‐sensitive mutants using the Gram‐positive transposon plasmid pMarA and adopting an enzyme digestion‐based inverse PCR strategy, we successfully identified insertion sites in most mutants, overcoming the limitations imposed by the high G + C content of the genome.

Functional analysis demonstrated that disrupting a plasmid‐encoded aminoglycoside phosphotransferase gene, *aph(3)*, eliminated streptomycin resistance. Although complementation and heterologous expression partially restored the resistant phenotype, the resistance levels remained lower than those observed in the wild‐type strain. Quantitative PCR revealed that this incomplete restoration was associated with *aph(3)* expression and the copy number of the plasmid pCM3, which harbors *aph(3)* (Figures S2B and S4). These findings indicate that the number of copies of both the plasmid and the gene critically influences *aph(3)* expression and streptomycin resistance. This is consistent with previous reports that the number of plasmid copies is tightly regulated to balance stability and host fitness^13^.

Mechanistically, this work provides the first evidence that streptomycin resistance in a plant‐pathogenic Gram‐positive bacterium is mediated by an APH(3)‐dependent phosphorylation pathway. Members of the APH(3) family catalyze the transfer of the γ‐phosphate from ATP to aminoglycoside antibiotics, resulting in antibiotic inactivation^14^. Phosphorylation of streptomycin produces streptomycin‐3′‐phosphate, with mono‐ and diphosphorylated forms predicted based on molecular weight calculations. This study expands our understanding of the strategies used by plant‐pathogenic bacteria to resist aminoglycoside antibiotics and highlights their relevance to recognized resistance mechanisms in clinical and environmental microbes.

Given the central role of pCM3 in mediating streptomycin resistance, understanding its origin and mobility is critical. *De novo* sequencing revealed that *aph(3)* is located on the previously uncharacterized plasmid, pCM3, which shares approximately 67% sequence homology with plasmids from *Curtobacterium* species, excluding the resistance gene itself. Annotation of pCM3 identified mobile genetic elements, including a MobC‐like relaxase, which is consistent with the potential for plasmid mobilization via HGT. Additionally, a 6.6 kb region containing genes encoding transposases, recombinase family proteins, and ATP‐binding proteins showed variable genomic localization across *C. michiganensis* strains, indicating extensive genetic rearrangements (Figure S1). Similar patterns have been reported in related *Clavibacter* species^15^, suggesting that genetic rearrangements may facilitate plasmid stabilization within the host.

From a broader perspective, the presence of antibiotic resistance genes (ARGs) in plant‐associated bacteria represents a growing concern for the One Health initiative. Although the dissemination of ARG has been extensively studied in soil, manure, and wastewater, relatively little attention has been paid to plant microbiomes^16^, ^17^, ^18^. *Curtobacterium* species, which commonly inhabit soil and plants and have occasionally been isolated from human clinical samples, may facilitate ARG exchange as environmental reservoirs^19^, ^20^. The identification of the *aph(3)* gene on a plasmid in *C. michiganensis* underscores the potential for HGT to connect environmental, plant, and human‐associated microbial communities.

In conclusion, we identified a novel plasmid, designated pCM3, in *C. michiganensis* strain TX‐0702. This plasmid confers streptomycin resistance through an *aph(3)*‐encoded phosphorylation mechanism. Our findings demonstrate that the number of plasmid copies strongly influences resistance levels and suggest that pCM3 was likely acquired through HGT from environmental bacteria. This study provides new insights into aminoglycoside resistance in plant‐pathogenic bacteria, highlighting the potential role of agricultural pathogens as reservoirs and vectors of ARGs. This emphasizes the need for enhanced surveillance and prudent antibiotic use in crop production systems.

## ETHICS STATEMENT

This study did not involve any human participant or animal subject.

## Supporting information

Supporting‐information file 1.

## Data Availability

The complete genome assembly data of *C. michiganensis* strain TX‐0702 have been deposited in the GenBank with the accession number PRJNA1230600.
