# Supplementary material for: Novel plasmid pCM3 harboring the aph(3) gene confers phosphorylation‐driven streptomycin resistance in Clavibacter michiganensis
Source: mLife. 2026 Jun 24;5(3):383–7. doi: 10.1002/mlf2.70091 (PMC13327613; doi:10.1002/mlf2.70091)
Supplement: Supplementary file 1 — Supporting‐information file 1. [file MLF2-5-383-s001.docx]

**Supporting information**

**Novel plasmid pCM3 harboring *aph(3)* gene confers phosphorylation-driven streptomycin resistance in *Clavibacter michiganensis***

**Xiaoli Xu^†1^, Cen Qian^†1, 2^, Zhigang Hao^1, 3^, Meng Xie^1^, Na Jiang^1^, Jianqiang Li^1^, Laixin Luo^1*^**

1. State Key Laboratory of Agricultural and Forestry Biosecurity; MARA Key Laboratory of Surveillance and Management for Plant Quarantine Pests, Beijing Key Laboratory of Seed Disease Testing and Control, College of Plant Protection, China Agricultural University, Beijing 100193, China

2. Shanghai Key laboratory of Agricultural Genetics and Breeding, Biotechnology Research Institute, Shanghai Academy of Agricultural Sciences, Shanghai 201106, China

3. Xinjiang Key Laboratory of Agricultural Biosafety；Institute of Plant Protection, Xinjiang Uygur Autonomous Region Academy of Agricultural Sciences, Urumqi 830091, Xinjiang, China

**Materials and methods**

**Bacterial strains, plasmids, and growth conditions**

The bacterial strains and plasmids used in this study are listed in **Table S3**. The wild-type *Clavibacter michiganensis* strain TX-0702, along with its mutant derivatives, was cultured in lysogeny broth (LB) at 28°C with shaking (150 rpm) or on LB agar (LBA, 1.8% [wt/vol] agar). *Escherichia coli* strains were grown in LB at 37°C with shaking (200 rpm) or on LBA. Selective antibiotics were added where necessary, at the following final concentrations: chloramphenicol, 10 µg/mL; kanamycin, 50 µg/mL; and ampicillin, 100 µg/mL.

**Construction of the loss-of-resistance mutant library**

To construct a transposon mutant library of *C. michiganensis* TX-0702, a modified transposon vector pMarA-Chl carrying a chloramphenicol resistance marker, was used. The pMarA-Chl plasmid was introduced into the competent TX-0702 cells via electroporation. Transformed cells were plated on LBA supplemented with 10 µg/mL chloramphenicol and incubated at 28°C for 5 d. The resulting colonies were subsequently selected on LBA containing both 10 µg/mL chloramphenicol and 50 µg/mL streptomycin to screen for chloramphenicol-resistant but streptomycin-sensitive insertion mutants. A total of 30 mutants were isolated and designated MT1-MT30.

Minimum inhibitory concentrations (MIC) were determined for all mutants to evaluate resistance levels. Mutants exhibiting MIC values below 20 µg/mL or those that failed to grow on LBA supplemented with 10 µg/mL chloramphenicol and 20 µg/mL streptomycin (LBA+Chl10+S20) were selected for further analysis.

**Determination of MIC of streptomycin against bacteria**

The MIC of streptomycin for different *C. michiganensis* strains was determined using an optimized microdilution assay . Briefly, 135 µL of LB medium containing bacterial suspension and 15 µL of streptomycin solution were dispensed into each well of a 96-well microtiter plate, with the final bacterial concentration was adjusted to 10^6^ CFU/mL. The first column served as a blank control (150 µL LB only). Streptomycin was serially dilutedb across the wells to achive final concentrations of 0, 1, 2, 4, 8, 16, 32, 64, 128, 256, and 512 µg/mL. Plates were sealed and incubated at 28°C with shaking for 48 hours. The optical density at 600 nm (OD600) was then measured using a multi-functional microplate reader (Infinite F200, Tecan, Switzerland). The MIC was defined as the lowest concentration at which a sharp decline in OD600 was odserved.

**Inverse PCR**

To identify transposon insertion sites, genomic DNA was extracted from selected streptomycin-sensitive mutants showing significant changes in MIC. Genomic DNA was digested with *Xho*I, *Sal*I, or *Ngo*MIV, which were selected based on their frequent recognition sites within the high-GC genome of TX-0702. The digested fragments were ligated to form self-circular DNA, which served as templates for inverse PCR. Primers targeting the 5' and 3' ends of the transposon were used to amplify the flanking genomic regions. PCR products were purified and sequenced. The obtained sequence contained both upstream and downstream genomic fragments interrupted by the transposon, enabling identification of the insertion sites and reconstruction of disrupted gene sequences.

***De novo* sequencing and data analysis**

Genomic DNA from TX-0702 was extracted using a Bacterial DNA Kit (Omega Bio-Tek, USA). DNA libraries for second-generation sequencing were constructed according to the manufacturer's instructions using the TruSeq DNA PCR-Free Library Preparation Kit and sequenced paired-end mode (PE150) on the Illumina NovaSeq 6000 platform. Low-quality raw reads (defined as regions covered by fewer than five reads) were removed prior to assembly. For third-generation sequencing, DNA libraries were constructed using EXP - NBD104/114 and SQK-LSK109 kits (Oxford Nanopore Technologies), and sequencing was performed on the PromethION platform. *De novo* assembly of long-read data was conducted using Canu, Flye-2.9, and Unicycler, and hybrid assembly was completed by incorporating Illumina reads to fill residual gaps.

Putative plasmid sequences were identified from assembled scaffolds based on size, coverage depth, and circularization status. Homology analysis was performed using BLASTn against the NCBI nucleotide database. Gene prediction was conducted using FGENESB (Softberry), and functional annotation was carried out using the NCBI Conserved Domain Database (CDD) and InterPro.

**Plasmid curing of *C. michiganensis* TX-0702**

Plasmid curing of *C. michiganensis* strain TX-0702 was conducted following the high-temperature stress method. Briefly, single colonies were streaked onto LBA plates and incubated at 33°C for 5 d under inverted conditions. Colonies were randomly selected and screened by colony PCR using plasmid-specific primers (**Table S4**) to assess the presence of the three native plasmids (pCM1, pCM2, and pCM3). For each round, one randomly chosen colony was subcultured and re-tested until loss of the target plasmids. Successfully cured strains were designated TX110 (lacking pCM3), TX010 (lacking pCM1 and pCM3), and TX000 (lacking all three plasmids).

**Disc diffusion assay for streptomycin sensitivity**

Streptomycin susceptibility of the plasmid-cured *C. michiganensis* strains was evaluated using the disc diffusion method. Overnight cultures were adjusted to 10⁶ CFU/mL and 100 μL of each suspension was spread evenly onto LBA. Sterile 6 mm filter paper discs were placed on the surface using sterile forceps. A volume of 5 μL ddH_2_O was applied to the disc on the left side as a negative control, while 5 μL of 1 mg/mL streptomycin solution was applied to the disc on the right. Plates were incubated at 28°C for 3 ~ 4 d. Zones of inhibition were measured to assess the antibacterial effect of streptomycin on each strain.

**Construction of the** ***aph(3)* deletion mutant and complementation strain**

The *aph(3)* single mutant was constructed in *C. michiganensis* TX-0702 via homologous recombination. Briefly, the upstream and downstream flanking regions of the *aph(3)* gene were PCR-amplified with the cmx chloramphenicol resistance cassette from plasmid pOKU9-cmBa using primers listed in **Table S4**. The amplified fragments were assembled into the *pEASY*-Blunt Simple Cloning Vector (TransGen Biotech, Beijing, China) to construct the suicide plasmid *pEASY*-*aph(3)*. The recombinant plasmid was electroporated into TX-0702. Transformants were selected on LBA supplemented with 10 µg/mL chloramphenicol and 50 µg/mL kanamycin. Colonies that were chloramphenicol-resistant and kanamycin-sensitive were screened by colony PCR and verified by Sanger sequencing (Tsingke, Beijing, China).

For complementation, the native *aph(3)* coding sequence and its upstream promoter region were amplified and cloned into a shuttle plasmid, then transformed into Δ*aph(3)*. Similarly, the expression cassette was introduced into TX-0702 and BT-0505 backgrounds to generate overexpression strains.

**RNA isolation and qPCR**

*C. michiganensis* strains were cultured to the exponential growth phase, and cells were harvested by centrifugation at 12,000 rpm. Total RNA was extracted using the TRNzol Universal Total RNA Extraction Kit (Tiangen Biotech, Beijing, China; DP424), and RNA purity and concentration were assessed via 1% (w/v) agarose gel electrophoresis and a NanoDrop spectrophotometer (Thermo Fisher Scientific, MA, USA).

For cDNA systhesis, 1 µg of RNA was reverse transcribed using the HiScript III 1st Strand cDNA Synthesis Kit (Vazyme, Nanjing, China). Quantitative PCR was performed in a 20 µL reaction volume containing 10 µL of 2× Taq Pro Universal SYBR qPCR Master Mix (Vazyme), 2 µL of cDNA template, 0.4 µL of each primer (10 µmol/L), and 7.2 µL sterile distilled water. The reaction was processed on an Applied Biosystems 7500 Fast Real-Time PCR System (Life Technologies, USA) with the following thermal cycling conditions: 95°C for 30 s, followed by 40 cycles of 95°C for 10 s, and 55°C for 30 s. Each reaction included three biological replicates and three technical replicates. Relative gene expression was normalized using three reference genes (*gyrB*, *bipA* and *gapA*). Relative transcription levels were calculated using the 2^−ΔΔCt^ method. All primers are listed in **Table S4.**

**Expression and purification of APH(3) protein**

The full-length *aph(3)* gene was cloned into the *pEASY*-Blunt E1 expression vector containing a 6×His tag and transformed into *E. coli* BL21 (DE3) cells. Positive transformants were cultured in LB medium with appropriate antibiotics at 37°C, 200 rpm until OD600 reached ~ 0.5. Protein expression was induced by 0.1 mM IPTG for 4 h. Cells were harvested by centrifugation at 8,000 rpm for 10 min and washed twice with PBS buffer. The cell pellet was resuspended in lysis buffer and subjected to sonication (150 W, 40 Hz, 3 s intervals, 30 min on ice). After centrifugation (8,000 rpm, 10 min), the supernatant was collected and loaded onto a Ni-affinity chromatography column pre-equilibrated with binding buffer. The column was washed twice with washing buffer and the APH(3) protein eluted with elution buffer on four 500 µL fractions. Protein purity was assessed via SDS-PAGE. The purified protein was concentrated and stored at −80°C until use.

**Phosphotransferase activity assay for streptomycin in vitro**

The phosphorylation of streptomycin *in vitro* was modified from previous studies ^51^ ^[30]^. Briefly, 1 mg of purified APH(3) protein was incubated with 1 mg of streptomycin in reaction buffer (40 mM Tris, 40 mM KCl, 10 mM MgCl_2_, 4 mM ATP) at 37°C for 2 h. A negative control lacking APH(3) was prepared in parallel. Five microliters of each reaction product were applied to 6 mm sterile filter paper discs and placed on LBA plates seeded with *C. michiganensis* (10^6^ CFU/mL). Growth inhibition zones were observed after incubation at 28°C for 48 h.

**LC-MS analysis of streptomycin phosphorylation products**

To confirm the phosphorylation of streptomycin, samples from the *in vitro* assay were analyzed using LC-MS. Chromatographic separation was performed using a Thermo Scientific™ Hypersil GOLD™ HILIC column (2.1 mm × 150 mm). The mobile phase is comprised of water (A) and acetonitrile (B). with the following gradient: 0-5 min, 5% A; 5-15 min, 5%-65% A; 15-20 min, 65%-90% A; 20-25 min, 90% A. The flow rate was 0.2 mL/min, and the injection volume was 15 μL.

Mass spectrometer was conducted in positive ESI mode. Ion source parameters included a nebulizer pressure of 30 psi. Nitrogen drying gas at 11 L/min and 325°C, and a capillary voltage of 4000 V. Streptomycin standards produced characteristic [M+H]^^+^ and [M+2H]^^2+^ peaks at m/z 582.27 and 291.64, respectively. Phosphorylated products were detected at m/z 662.24 ([M+H]^^+^) and 331.62 ([M+2H]^^2+^), confirming the addition of a phosphate group.

**Table S1** The minimum inhibitory concentration (MIC) of streptomycin against 30 transposon inserted mutants of *C. michiganensis* TX-0702.

| **Strain** | **MIC (μg/mL)** | **Strain** | **MIC (μg/mL)** | **Strain** | **MIC (μg/mL)** | **Strain** | **MIC (μg/mL)** |
| --- | --- | --- | --- | --- | --- | --- | --- |
| MT1 | 4 | MT2 | 4 | MT3 | 4 | MT4 | 2 |
| MT5 | 2 | MT6 | 4 | MT7 | 4 | MT8 | 16 |
| MT9 | 16 | MT10 | 4 | MT11 | 16 | MT12 | 4 |
| MT13 | 16 | MT14 | 2 | MT15 | 16 |  |  |
| MT16 | 32 | MT17 | 32 | MT18 | 32 | MT19 | 32 |
| MT20 | 32 | MT21 | 128 | MT22 | 64 | MT23 | 128 |
| MT24 | 64 | MT25 | 64 | MT26 | 64 | MT27 | 64 |
| MT28 | 64 | MT29 | 64 | MT30 | 64 |  |  |

**Table S2** Transposon insertion site information of reduced streptomycin resistance mutants of C. michiganensis TX-0702.

| **Strain** | **Gene ID** | **Gene function** |
| --- | --- | --- |
| MT1 | Cm_01801 | ABC-2 family transporter protein |
| MT2, MT3 | Cm_01616 | ATP-dependent RecD-like DNA helicase |
| MT13 | Cm_01667 | Membrane protein insertase MisCA precursor |
| MT15 | Cm_00227 | hypothetical protein |
| MT5, MT7, MT8, MT11, MT12, MT14, MT19 | / | / |

“/” indicates that insertion site did not align reliably with any sequences in the TX0702 resequencing database or the NCBI database.

**Table S3** Strains and plasmids used in the current study.

| **Strain Name** | **Genotype** | **Reference** |
| --- | --- | --- |
| ***Escherichia coli*** |  |  |
| *Trans*5*α* | F^-^ φ80d *lac*ZΔM15 Δ(*lac*ZYA-*arg*F) U169 *end* A1 *recA*1 *hsd*R17(r_k_ ^-^ ,m_k_ ^+^ ) *sup*E44λ-*thi*-1 *gyr*A96 *rel*A1 *pho*A | TransGen Biotech |
| BL21 (DE3) | F^-^ *omp*T *hsd*S_B_ (r_B_ ^-^ , m_B_ ^-^ ) *gal* *dcm* (DE3) | TransGen Biotech |
| ***Clavibacter michiganensis*** | | |
| TX-0702 | *C. michiganensis* wild-type | Laboratory |
| BT-0505 | *C. michiganensis* streptomycin-sensitive strain | Laboratory |
| TX-0702+pHN216-EV | *C. michiganensis* TX-0702 containing pHN216, Neo^R^ | The current study |
| Δ*aph(3)* | *C. michiganensis aph(3)* deletion mutant, TX-0702 derivative, Cm^R^ | The current study |
| Δ*aph(3)*+pHN216-EV | *C. michiganensis* Δ*aph(3)* containing pHN216, Neo^R^ | The current study |
| Δ*aph(3)*::*aph(3)* | Complementation isolate: Δ*aph(3)* containing pCM*aph(3)*, Cm^R^ Neo^R^ | The current study |
| TX-0702+*aph(3)* | *C. michiganensis* TX-0702 containing pCM*aph(3)*, Neo^R^ | The current study |
| BT-0505+pHN216-EV | *C. michiganensis* BT-0505 containing pHN216, Neo^R^ |  |
| BT-0505+*aph(3)* | *C. michiganensis* BT-0505 containing pCM*aph(3)*, Neo^R^ | The current study |
| **Plasmid** |  |  |
| p*EASY*-Blunt Simple | Simple cloning vector, Neo^R^ | TransGen Biotech |
| pHN216 | *E. coli*-*Clavibacter* shuttle vector. Gen^R^ , Neo^R^ | [1] |
| p*EASY*-*aph(3)* | p*EASY*-Blunt Simple containing *C. michiganensis* *aph(3)/cmx* In-Fusion fragment used for homologous recombination to produce Δ*aph(3)*, Km^R^ | The current study |
| pCM*aph(3)* | pHN216 containing the *C. michiganensis* *xysB* gene and its native promoter, NeoR | The current study |
| pEBE*aph(3)* | Vectors for expression of APH(3) | The current study |

**Table S4** Primers used for the PCR analysis conducted in the current study.

| **Primer** | **Sequence (5’→3’)** | **Target** | **Refence** |  |  |  |
| --- | --- | --- | --- | --- | --- | --- |
| **qRT-PCR** | | | | | |  |
| gyrB-RT-F | GGACAGCACATCACGACCC | *gyrB* | [2] |  |  |  |
| gyrB-RT-R | CCTTCGGCATCTTCTTCCC |  |  |  |  |  |
| bipA-RT-F | GGGTGCTGGTCGTCGTA | *bipA* | [2] |  |  |  |
| bipA-RT-R | CGAGCCGCTGTTCAAG |  |  |  |  |  |
| gapA-RT-F | TTGACCTGGTTGCCGATGAC | *gapA* | [2] |  |  |  |
| gapA-RT-R | TCAACGACCCGCACTCCTC |  |  |  |  |  |
| q-aph-F2 | GCAGTCACACTCGCTCACTC | *aph(3)* | The current study |  |  |  |
| q-aph-R2 | GTTCCTCGTGCCAGTCAAGT |  |  | |  |  |
| **pMarA-Chl** | | | | |  |  |
| pMARa-F | GTCTCCGGGGGGATCCTACCTAGATTTAGATGTCTAAAAAG | pMarA-Chl vector | The current study |  |  |  |
| pMARa-R | CGTTAAGTATTTCACGGGGATCCTTATTATTTCCTTCCTCT |  |  |  |  |  |
| Cmx-F | GGAAGGAAATAATAAGGATCCCCGTGAAATACTTA | *cmx* gene for pMarA-Chl vector | The current study |  |  |  |
| Cmx-R | TAAATCTAGGTAGGATCCCCCCGGAGA |  |  |  |  |  |
| ZZZ-F | TGCAGTAACAGGTTGGCTGATAAGTCCCCGGTCTG | Identification of the transposon region of the pMarA-Chl vector | The current study |  |  |  |
| ZZZ-R | CTATGACCATGATTACGCCAAGCTTGCATGCCTGCA |  |  |  |  |  |
| SuK-F | TTGATCAGCGTGTCATGGTCGG | SuK sequence | The current study |  |  |  |
| SuK-R | CCACTCCTCCGATGATCACTGTTC |  |  |  |  |  |
| **Plasmid curing** | | | |  |  |  |
| pCM1_2ParA-F | GTGCTCCTTGTTCATCTTCC | *parA* (replication initiation, pCM1) | The current study |  |  |  |
| pCM1_2ParA-R | ATCCGCTAGTGTCGTCTTGC |  |  |  |  |  |
| pCM2_25ParA-F | GGACTACGACACGATCATCG | *parA* (replication initiation, pCM2) | The current study |  |  |  |
| pCM2_25ParA-R | TACATTCCTACCTAGTTACG |  |  |  |  |  |
| pCM3_1parB-F | CGTACCTGACCAAGGAGTTC | *parB* (replication initiation, pCM3) | The current study |  |  |  |
| pCM3_1parB-R | GAAGTACTTCTCCACCCGGA |  |  |  |  |  |
| ***aph(3)* gene editing** | | | | |  |  |
| I-*aph(3)*-up-F | GCTGCCCTTGGGCGAACCCCTTCCCCTG | *aph(3)* upsteam for T- *aph(3)* | The current study |  |  |  |
| I-*aph(3)*-up-R | CGGGGATCCGAACTAGGGGGACCCATGGC |  |  |  |  |  |
| I-*aph(3)*-down-F | GGGGGATCCCGCGACTCGCTCAGCTGTC | *aph(3)* downsteam for T- *aph(3)* | The current study |  |  |  |
| I-*aph(3)*-down-R | ATTGAAGCTGCCCTTCCACTCCTCCGATGATCACTG |  |  |  |  |  |
| I-CMX-*aph(3)*-F | GGGTCCCCCTAGTTCGGATCCCCGTGAAATACTTAACG | *cmx* for T- *aph(3)* | The current study |  |  |  |
| I-CMX-*aph(3)*-R | CGAGTCGCGGGATCCCCCCGGAGACGGG |  |  |  |  |  |
| *aph(3)*-T-F | GCACGGCCTTCACGTGGTCG | Identification of *aph(3)* knockout transformants | The current study |  |  |  |
| *aph(3)*-T-R | TCGAGTGCCGTACCCGCGG |  |  |  |  |  |
| 216-*aph*-F | GCTTGCGGCAGCGTGATTGATCAGCGTGTCATGGT | *aph(3)* gene gene containing native promoter for pCM*aph(3)* | The current study |  |  |  |
| 216-*aph*-R | CGGTACTTGGGTCGATCCACTCCTCCGATGATCA |  |  |  |  |  |
| **Protein purification** | | | | | | |
| pEBE1-CW-F | AAGGGCCAATTCGAGCTCAAC | *aph(3)* gene for pEBE*aph(3)* | The current study |  |  |  |
| pEBE1-CW-R | CATCTTGTTCAATCATACTCTTCCTTTTTC |  |  |  |  |  |
| pEBE1-GJ-F | CGAGTTCTTCTGACTGTCAGACCAAGTTTACTC | linearised p*EASY* Blunt E1 | The current study |  |  |  |
| pEBE1-GJ-R | AAGGGCCAATTCGCTAGCC |  |  |  |  |  |
| pEBE1-Kan-F | GGAAGAGTATGATTGAACAAGATGGATTGCAC | Identification of pEBE*aph(3)* | The current study |  |  |  |
| pEBE1-Kan-R | CTTGGTCTGACAGTCAGAAGAACTCGTCAAG |  |  |  |  |  |
| PGEX-F | TGACCCGGGTCGACTCGAG | linearised PGEX | The current study |  |  |  |
| PGEX-R | GAATACTGTTTCCTGTGTGAAATTG |  |  |  |  |  |


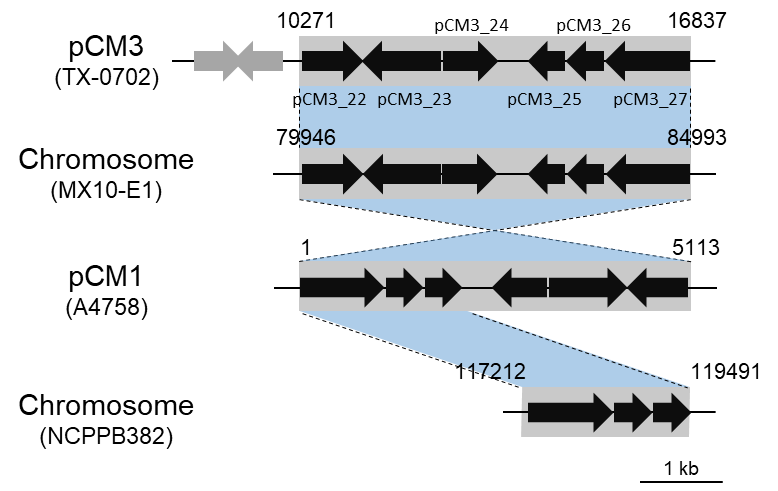


**Figure S1** Schematic alignment of conserved genomic regions among sequences of plasmid pCM3 in *Clavibacter michiganensis* TX-0702 and related sequences in other *C. michiganensis* strains. Sequences of strains MX-10E-1, A4758 and NCPPB382 were retrieved from NCBI. Arrows indicate the orientation and extent of genetic sequences. Light blue shaded regions denote homologous regions, and the average identity is >88%.


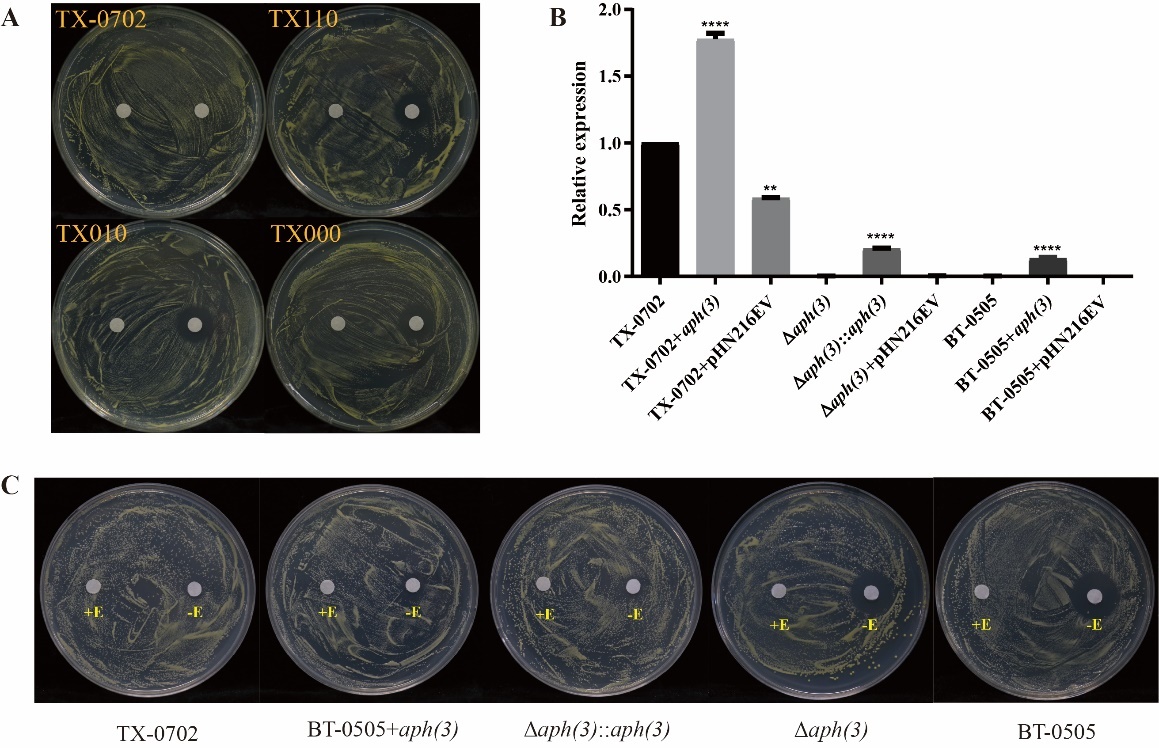


**Figure S2** Disc diffusion assays confirm the contribution of *aph(3)* to streptomycin resistance. A) Streptomycin sensitivity of plasmid-cured strains. Strains were diluted to 10^6^ CFU/mL, and plated on LBA, respectively. Sterilized filter paper discs were placed on each plate, with 5 μL of ddH_2_O applied to the left disc and streptomycin to the right disc. B) Expression levels of *aph(3)* gene in different *C. michiganensis* strains. Data are shown as mean ± SD (n = 3); ** and **** indicate significant differences compared with TX-0702 (one-way ANOVA, *p* < 0.01and *p* < 0.0001). C) Streptomycin sensitivity of *C. michiganensis* strains with different levels of *aph(3)* expression. Discs contained streptomycin with or without APH(3) pretreatment.


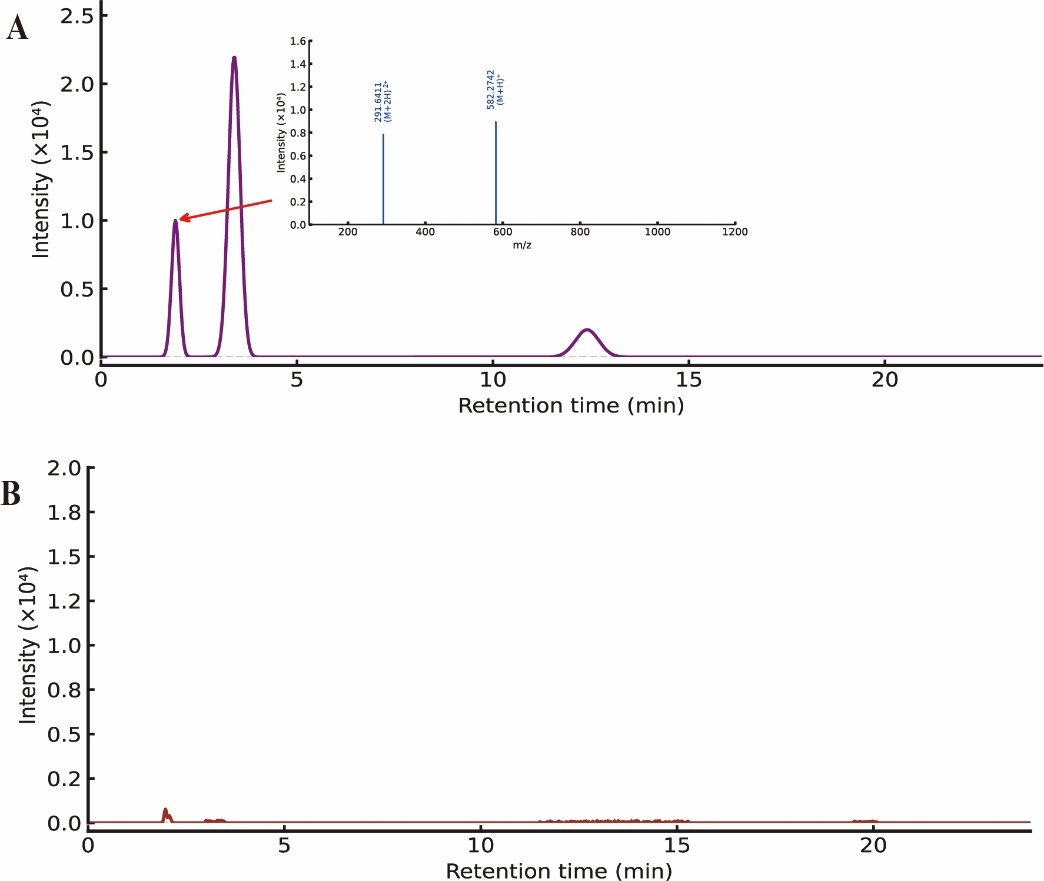


**Figure S3** Detection of streptomycin and its phosphorylated derivatives with distilled water-treated control by LC-MS. A) LC-MS detection of streptomycin in negative control. Peaks at m/z 582.27 and the doubly charged ion [M+2H]^2+ at m/z 291.64 correspond to native treptomycin ions. B) LC-MS detection of phosphorylated streptomycin in negative control. Phosphorylated streptomycin peaks were absent.

**Figure S4** Determination of the absolute copy number of plasmid pCM3 in TX-0702 and Δ*aph(3)*::*aph(3)* by qPCR. Each strain normalized using its own single-copy housekeeping gene *bipA* to obtain the absolute copies of the pCM3 plasmid. Error bars indicate standard deviations from three independent experiments; and asterisks **** significant differences (*p <* 0.0001) as determined by *t-*test.

**References**

[1] M.J. Laine, H. Nakhei, J. Dreier, K. Lehtilä, D. Meletzus, R. Eichenlaub, et al., Stable transformation of the gram-positive phytopathogenic bacterium *Clavibacter michiganensis* subsp. *sepedonicus* with several cloning vectors, Appl. Environ. Microbiol. 62 (1996) 1500–1506. doi:10.1128/aem.62.5.1500-1506.1996.

[2] N. Jiang, Q. Lyu, S. Han, X. Xu, R.R. Walcott, J. Li, et al., Evaluation of suitable reference genes for normalization of quantitative reverse transcription PCR analyses in *Clavibacter michiganensis*, MicrobiologyOpen 8 (2019) e928. doi:10.1002/mbo3.928
